# Integrated multi-omics analysis reveals distinct molecular features of key stages in cotton fiber development in *Gossypium hirsutum*

**DOI:** 10.3389/fpls.2026.1850466

**Published:** 2026-08-13

**Authors:** Zhaolong Gong, Shengmei Li, Lurong Xu, Shuaishuai Qian, Ni Yang, Haihong Chen, Fenglei Sun, Shiwei Geng, Yajun Liang, Xueyuan Li, Juyun Zheng, Junduo Wang

**Affiliations:** 1 Xinjiang Key Laboratory of Cotton Genetic Improvement and Intelligent Production/Xinjiang Cotton Technology Innovation Center, Cotton Research Institute of Academy of Agricultural Sciences of Xinjiang Uyghur Autonomous Region (National Cotton Engineering Technology Research Center), Urumqi, Xinjiang, China; 2 College of Biotechnology, Xinjiang Agricultural Vocational and Technical University, Changji, China

**Keywords:** fiber development, flavonoid biosynthesis, *Gossypium hirsutum*, multi-omics, phenylpropanoid metabolism

## Abstract

**Introduction:**

Cotton fiber development is a critical biological process underlying fiber quality, and its regulation involves multiple molecular layers, including transcription, translation, and metabolism.

**Methods:**

To systematically elucidate the molecular basis of fiber development in *Gossypium hirsutum*, we selected two cultivars with contrasting fiber quality, Sumian 11 (SM11) and Yumian 5 (YM5), and conducted integrated transcriptomic, proteomic, and metabolomic analyses of fibers collected at 15, 20, 25, and 30 days post anthesis (DPA).

**Results:**

As fiber development progressed, the numbers of differentially expressed genes, proteins, and accumulated metabolites increased in both cultivars, indicating extensive molecular reprogramming during the later stages of development. Cross-omics comparisons identified plant hormone signal transduction, starch and sucrose metabolism, phenylpropanoid biosynthesis, flavonoid biosynthesis, cutin, suberin and wax biosynthesis, and ABC transporters as core pathways commonly involved in fiber development. SM11 exhibited stronger enrichment of phenylpropanoid metabolism, cytochrome P450, and MAPK signaling, whereas YM5 showed more pronounced enrichment of ribosome-related processes, fatty acid elongation, and nitrogen metabolism. Proteomic and metabolomic analyses further confirmed substantial differences between the two cultivars in phenylpropanoid and flavonoid metabolism, sugar metabolism, and lipid metabolism. Integrated multi-omics analysis further demonstrated that phenylpropanoid and flavonoid biosynthesis constitute key coordinated modules across the three omics layers. Metabolites such as taxifolin, dihydromyricetin, and sinapaldehyde were closely associated with candidate genes and proteins, together forming an interconnected regulatory network.

**Discussion:**

These findings provide mechanistic insights into the molecular regulation of cotton fiber development and identify candidate molecular targets for fiber quality improvement in *G. hirsutum*.

## Introduction

Cotton is one of the most important natural fiber crops worldwide (Li X, et al., 2026). Cotton fibers are highly specialized single cells that differentiate from the epidermal cells of the ovule outer integument, and their development is a major determinant of key fiber quality traits, including length, strength, and fineness (Jan et al., 2022; Zhao et al., 2026). Owing to its unique single-cell nature, cotton fiber has been widely recognized as an ideal model system for investigating fundamental biological processes such as cell elongation, cell wall remodeling, and cellulose biosynthesis (Jareczek et al., 2023). Previous studies have shown that cotton fiber development is a dynamic and continuous process with partially overlapping phases, which can be broadly divided into five stages: initiation, rapid elongation, transition/primary cell wall remodeling, secondary cell wall biosynthesis, and maturation (Li G, et al., 2026). Fiber initiation generally occurs from approximately −3 to 3 days post-anthesis (DPA), followed by rapid elongation from 3 to 16 DPA. The transition stage, during which primary cell wall remodeling becomes prominent, typically spans 16 to 20 DPA, after which fibers enter secondary cell wall biosynthesis from approximately 20 to 40 DPA. Maturation then occurs during the final stage, from approximately 40 to 50 DPA (Jareczek et al., 2023; Bai and Scheffler, 2024).

During cotton fiber development, the transition from rapid elongation to secondary cell wall deposition represents a critical window that strongly influences final fiber quality (Thenveettil et al., 2025). High-temporal-resolution transcriptomic studies have shown that a major developmental shift from elongation to secondary wall biosynthesis occurs at approximately 16–20 DPA in *Gossypium hirsutum* fibers, whereas rapid dry matter accumulation and large-scale cellulose production begin around 20–25 DPA (Grover et al., 2025). These observations highlight this interval as a pivotal stage for cell wall remodeling and fiber quality formation. Further molecular investigations have demonstrated that *GhCesA4*, *GhCesA7*, and *GhCesA8* assemble into a fiber-specific cellulose synthase supercomplex during secondary wall formation (Chen et al., 2025a; Hussain et al., 2026). Loss of any one of these components abolishes the complex and severely impairs secondary wall deposition, indicating that the coordinated temporal expression of cell wall-related genes and their encoded proteins provides an important molecular basis for fiber quality formation (Wang et al., 2025). In addition, integrated transcriptomic and metabolomic analyses of fiber samples collected from two cotton genotypes at 10–28 DPA identified a relatively independent transitional stage of cell wall remodeling prior to the onset of secondary wall cellulose biosynthesis and further suggested that lignification-related processes are transcriptionally repressed during secondary wall formation (Tuttle et al., 2015). Studies on naturally colored cotton have likewise shown that the phenylpropanoid pathway is not merely a peripheral branch unrelated to fiber development; in green cotton, genes associated with the biosynthesis of caffeic acid derivatives, lignin, and lignans are significantly induced at 10 and 15 DPA (Li et al., 2020).

With advances in high-throughput sequencing and mass spectrometry, multi-omics approaches have become powerful tools for dissecting the molecular mechanisms underlying plant growth and development (Song et al., 2026; Kundu and Tanti, 2026). Previous studies have shown that integrative analyses combining transcriptomics, proteomics, phosphoproteomics, and genetic mapping in a fiberless cotton mutant highlighted amino sugar and nucleotide sugar metabolism, fatty acid metabolism, and flavonoid biosynthesis as key processes associated with fiber initiation (Ma et al., 2016). Time-series transcriptomic analyses based on continuous sampling of TM-1, Hai7124, 3-79, and related materials identified 15–25 DPA as a critical period for fiber quality formation and highlighted candidate genes associated with secondary cell wall biosynthesis and phytohormone regulation (Zhang et al., 2022). More recently, integrated spatial transcriptomic, single-cell transcriptomic, and spatial metabolomic analyses have generated spatiotemporal molecular landscapes of cotton fiber initiation and early development (Sun et al., 2025). In parallel, other studies integrating glycomics, transcriptomics, and proteomics at high temporal resolution analyzed fibers from *G. hirsutum* and *G. barbadense* collected daily from 6 to 25 DPA, revealing substantial differences in cell wall polysaccharide composition and the dynamics of associated glycosyltransferases (Swaminathan et al., 2026). Meanwhile, integrated transcriptomic and metabolomic analyses of naturally brown cotton fibers have shown that 15–20 DPA represents a key developmental window characterized by high activity of flavonoid/proanthocyanidin metabolism and accumulation of related regulatory factors (Wang et al., 2022). Collectively, these findings underscore the value of multi-omics integration in elucidating the molecular basis of fiber quality formation.

Although substantial progress has been made in understanding cotton fiber initiation, elongation, transition, and secondary wall formation, the critical 15–30 DPA window remains insufficiently characterized, especially by studies integrating transcriptomic, proteomic, and metabolomic data across contrasting *G. hirsutum* genotypes. This period encompasses the termination of fiber elongation, cell wall remodeling, and rapid secondary wall deposition, all of which are closely linked to fiber quality formation. In particular, how cell wall formation and phenylpropanoid metabolism are coordinately regulated, and how their interplay contributes to fiber quality variation, remains unclear at the multi-omics level. To address this gap, we selected two *G. hirsutum* genotypes with contrasting fiber traits and performed integrated transcriptomic, proteomic, and metabolomic analyses of fibers collected at 15, 20, 25, and 30 DPA. Our objectives were to define the molecular differences between the two genotypes during this critical developmental window, identify candidate genes, proteins, and metabolites associated with fiber quality variation, and provide a basis for future functional validation.

## Materials and methods

### Plant materials

Two *G. hirsutum* cultivars exhibiting marked differences in fiber quality, Yumian 5 (YM5, high fiber quality) and Sumian 11 (SM11, low fiber quality), were used in this study. The plant materials were obtained from and maintained by the Cotton Research Institute, Xinjiang Academy of Agricultural Sciences (**Supplementary Figure 1**). SM11 is an *G. hirsutum* cultivar originating from Jiangsu Province, China, and adapted to the Yangtze River cotton-growing region. It was reportedly released in 1997 and was derived from the cross Xiangmian 10 × CAdcs-2-81. Its genetic background therefore primarily represents germplasm from the Yangtze River cotton-growing region of China, with genetic contribution from the CAdcs-2–81 line. YM5 is an *G. hirsutum* cultivar originating from Henan Province, China, and adapted to the Yellow River cotton-growing region. It was reportedly released in 1989 and was developed from the cross CRI 10 × (Heishanmian 1 × Xinxiang 1). Both cultivars were grown during the 2025 growing season at the Manas Experimental Station of the Xinjiang Academy of Agricultural Sciences, Manas, Xinjiang, China (44.30°N, 86.25°E). The field experiment was arranged in a randomized block design, with a row length of 10 m, row spacing of 0.35 m, and plant spacing of 0.10 m. Irrigation, fertilization, and pest and disease control were carried out according to standard local agronomic practices. Flowers that opened on the same day were tagged and designated as 0 DPA. Developing fiber samples were collected at 15, 20, 25, and 30 DPA. To minimize the effects of boll position on fiber development, all samples were collected from normally developing bolls located at the same fruiting branch position, node position, and canopy level. For each cultivar at each developmental stage, at least three independent biological replicates were collected, and each replicate consisted of pooled fibers from at least five uniformly growing, healthy plants. After harvest, all samples were immediately frozen in liquid nitrogen and stored at −80 °C until further use for transcriptome sequencing (n = 3), proteomic analysis (n = 3), metabolomic analysis (n = 6), and qRT-PCR validation (n = 3).

### RNA-seq analysis

Cotton fiber samples were transported on dry ice to OE Biotech Co., Ltd. (Shanghai, China) for total RNA extraction, library construction, and sequencing. Total RNA was extracted using a plant total RNA extraction kit optimized for polysaccharide- and polyphenol-rich tissues according to the manufacturer’s instructions. RNA concentration and purity were measured using a NanoDrop spectrophotometer, and RNA integrity was assessed using an Agilent 2100 Bioanalyzer. Only RNA samples meeting the following quality criteria were used for library construction: OD260/280 = 1.8-2.2, OD260/230 ≥ 2.0, and RNA integrity number (RIN) ≥ 7.0. Poly(A) mRNA was enriched using Oligo(dT) magnetic beads and randomly fragmented in fragmentation buffer. The fragmented mRNA was then used as the template for first- and second-strand cDNA synthesis. After purification, double-stranded cDNA was subjected to end repair, 3′ adenylation, adapter ligation, fragment size selection, and PCR amplification to generate sequencing libraries with an insert size of approximately 370–420 bp. After library quality assessment, paired-end sequencing (150 bp) was performed on the Illumina HiSeq 2500 platform.

Raw sequencing data were processed using fastp to remove adapter sequences, low-quality reads, and reads containing a high proportion of ambiguous bases (N), yielding clean reads (Chen et al., 2018). The clean reads were then aligned to the *Gossypium hirsutum* TM-1 reference genome (TM-1 v2.1) using HISAT2 (Hu et al., 2019; Kim et al., 2019). Based on the alignment results, transcript assembly and expression quantification were performed using StringTie, and gene expression abundance together with raw count values for each sample was obtained (Pertea et al., 2015). Differential expression analysis was conducted using the DESeq2 package, with genes showing |log2 fold change| ≥ 1 and a false discovery rate (FDR) < 0.01 defined as differentially expressed genes (DEGs) (Love et al., 2014). The identified DEGs were further subjected to Gene Ontology (GO) and Kyoto Encyclopedia of Genes and Genomes (KEGG) enrichment analyses using the clusterProfiler package (version 4.0) in R, with adjusted P < 0.05 used as the threshold for significant enrichment (Wu et al., 2021).

### Metabolite extraction and quantification

Cotton fiber samples (100 mg) were ground into a fine powder in liquid nitrogen, and metabolites were extracted with 500 μL of pre-chilled 80% methanol in water. After vortexing, the samples were incubated on ice for 5 min and centrifuged at 15,000 × g for 20 min at 4 °C. The supernatant was collected and diluted with MS-grade water to a final methanol concentration of 53%, followed by a second centrifugation at 15,000 × g for 20 min at 4 °C. The resulting supernatant was used for subsequent liquid chromatography-tandem mass spectrometry (LC-MS/MS) analysis. Metabolite profiling was performed on an LC-MS/MS platform. The mass spectrometry parameters were as follows: Curtain Gas, 35 psi; Collision Gas, medium; IonSpray Voltage, 5500 V in positive ion mode and −4500 V in negative ion mode; ion source temperature, 550 °C; and Ion Source Gas 1 and Ion Source Gas 2, both set at 60 psi. Metabolites were detected in multiple reaction monitoring (MRM) mode using the in-house database novoDB established by Novogene. Metabolite identification was performed based on precursor ion (Q1), product ion (Q3), retention time (RT), declustering potential (DP), and collision energy (CE). Relative quantification was based on the peak area of the corresponding Q3 ion. Raw mass spectrometry data were processed using SCIEX OS software (version 1.4) for peak detection, integration, correction, and filtering. The relevant parameters were set as follows: minimum peak height, 500; signal-to-noise ratio, 5; and smoothing points, 1. The integrated product ion peak area of each metabolite within the corresponding retention time window was used to represent its relative abundance, generating qualitative and relative quantitative metabolite profiling data.

### Metabolomic analysis

The identified metabolites were functionally annotated against the KEGG, HMDB, and LIPID MAPS databases (Kanehisa et al., 2025; Wishart et al., 2022; Conroy et al., 2024). Raw data were processed using metaX for normalization, transformation, and quality control, followed by principal component analysis (PCA) and partial least squares-discriminant analysis (PLS-DA) to assess metabolic variation among samples and identify discriminatory metabolites (Wen et al., 2017). Variable importance in projection (VIP) values for individual metabolites were obtained from the PLS-DA model, and model robustness was further evaluated by permutation testing. Differences in metabolite abundance were assessed using Student’s t-test, and fold change (FC) values were calculated simultaneously. Metabolites with VIP > 1, adjusted P < 0.05, and FC ≥ 2 or FC ≤ 0.5 were defined as differentially accumulated metabolites (DAMs). Clustered heatmaps of DAMs were generated using the pheatmap package in R after Z-score normalization of metabolite abundance data. KEGG pathway enrichment analysis of DAMs was subsequently performed to characterize metabolic reprogramming associated with fiber development across different cultivars and developmental stages.

### Protein extraction, digestion, and DIA-LC-MS/MS analysis

Cotton fiber samples were thoroughly ground into a fine powder under low-temperature conditions, and total proteins were extracted using lysis buffer followed by ultrasonic disruption on ice. The lysates were centrifuged at 12,000 × g for 20 min at 4 °C, and the supernatants were collected and centrifuged once more to remove insoluble impurities. Protein concentration was determined using the bicinchoninic acid (BCA) assay. For reduction, dithiothreitol (DTT) was added to a final concentration of approximately 5 mM, and the samples were incubated at 55 °C for 30 min. Subsequently, iodoacetamide (IAA) was added to a final concentration of approximately 10 mM, and alkylation was performed at room temperature for 15 min in the dark. Proteins were then precipitated by adding 4–6 volumes of pre-chilled acetone and incubating at −20 °C for at least 4 h or overnight. The protein precipitates were collected by centrifugation, washed with pre-chilled acetone, briefly air-dried, and resuspended in digestion buffer. Based on the protein quantification results, equal amounts of total protein from each sample were adjusted to the same final volume. Trypsin was added at a protein-to-enzyme ratio of 50:1 (w/w), and digestion was carried out at 37 °C for 4 h, followed by additional trypsin supplementation for overnight digestion. After digestion, formic acid was added to adjust the pH to 1–3 and terminate the reaction. The resulting peptides were desalted using C18 reverse-phase solid-phase extraction columns and lyophilized for storage.

For LC-MS/MS analysis, 4 μg of peptides from each sample was mixed with 0.8 μL of iRT standard and separated using an EASY-nLC 1200 nano-ultra-high-performance liquid chromatography system. A self-packed trap column (4.5 cm × 75 μm, 3 μm) and a self-packed analytical column (15 cm × 150 μm, 1.9 μm) were used for peptide separation. Mobile phase A consisted of 0.1% formic acid in water, and mobile phase B consisted of 80% acetonitrile containing 0.1% formic acid. The separated peptides were subsequently analyzed on a Q Exactive HF-X mass spectrometer equipped with a NanosprayFlex ESI ion source in data-independent acquisition (DIA) mode (Lou and Shui, 2024). The mass spectrometry parameters were set as follows: spray voltage, 2.1 kV; capillary temperature, 320 °C; full MS scan range, m/z 350-1500; full MS resolution, 60,000; C-trap maximum capacity, 5 × 10^5; and maximum injection time, 20 ms. For MS/MS scans, the resolution was set to 30,000, the C-trap maximum capacity to 1 × 10^6^, and the normalized higher-energy collisional dissociation (HCD) collision energy to 27%.

### Proteomic data analysis

Raw mass spectrometry data were processed using DIA-NN for database searching and DIA-based protein quantification (Demichev et al., 2020). Protein identification was performed against the Gossypium hirsutum TM-1 reference protein database used in the transcriptomic analysis, supplemented with a common contaminant protein database to minimize false-positive identifications. Trypsin was specified as the digestion enzyme. Carbamidomethylation of cysteine [Carbamidomethyl (C)] was set as a fixed modification, whereas methionine oxidation [Oxidation (M)] and protein N-terminal acetylation [Acetyl (Protein N-term)] were set as variable modifications. The FDR was controlled at <1% at both the peptide and protein levels. Differentially accumulated proteins (DAPs) were defined as those with FC > 1.2 or FC < 0.83 and FDR < 0.05. Functional annotation and enrichment analyses were performed to characterize the biological features of the identified proteins and DAPs. Annotation was conducted against the UniProt, STRING v12, KEGG and InterPro databases (Szklarczyk et al., 2023; UniProt, 2018). GO, KEGG, and other pathway annotation results for DAPs were subsequently used for functional classification and biological interpretation. Statistical analyses and data visualization were performed using Python 3.9.16 and R 4.2.2.

### Integrated multi-omics analysis

To systematically dissect coordinated molecular changes during cotton fiber development, DEGs, DAPs, and DAMs were subjected to integrated multi-omics analysis. KEGG pathway annotation and enrichment analyses were first performed separately for each class of differential molecules, and candidate genes, proteins, and metabolites commonly enriched in the same pathways were identified (Kanehisa et al., 2025). Based on these shared pathways, correlation analyses were then conducted among the differential molecules. After standardization of gene expression, protein abundance, and metabolite relative abundance data, Pearson’s correlation coefficients were calculated using the cor function in R, and statistical significance was evaluated accordingly. Significant correlations were defined as q < 0.05 and |r| > 0.90. Multiple testing was corrected using the Benjamini-Hochberg (BH) method to control the FDR (Benjamini and Hochberg, 1995). Only molecular pairs that met these statistical criteria and were located in commonly and significantly enriched KEGG pathways were retained for network visualization and key node identification. The resulting networks were visualized in Cytoscape (Shannon et al., 2003).

### qRT-PCR

To validate the reliability of the RNA-seq data, 10 DEGs were selected for qRT-PCR analysis. Total RNA samples from the same batch used for transcriptome sequencing were reverse-transcribed into first-strand cDNA using a reverse transcription kit according to the manufacturer’s instructions. qRT-PCR was performed using 2 × SYBR Green qPCR Master Mix. Gene-specific primers were designed using NCBI Primer-BLAST and synthesized by Sangon Biotech Co., Ltd. (Shanghai, China) (**Supplementary Table 1**). Each reaction was carried out in a total volume of 20 μL containing 10 μL of 2 × SYBR Green qPCR Master Mix, 0.4 μL each of forward and reverse primers, 2 μL of cDNA template, and nuclease-free water to a final volume. Amplification was performed on a Bio-Rad CFX96 Real-Time PCR System (Bio-Rad Laboratories, Hercules, CA, USA) using the following program: 95 °C for 30 s, followed by 40 cycles of 95 °C for 10 s and 60 °C for 30 s. *GhUBQ7* was used as the internal reference gene, and relative gene expression levels were calculated using the 2^−ΔΔCt^ method (Livak and Schmittgen, 2001). Three biological replicates and three technical replicates were included for each sample.

## Results

### Quality and reproducibility assessment of transcriptomic sequencing data

To characterize the transcriptional dynamics underlying fiber development in cotton materials with contrasting fiber quality, RNA-seq was performed on 24 fiber samples collected from YM 5 and SM 11 at 15, 20, 25, and 30 DPA. A total of 155.51 Gb of clean data was obtained, with 5.82-7.02 Gb generated per sample. Sequencing quality was high across all samples, with Q30 values ranging from 96.98% to 97.95% and an average GC content of 44.01%. Alignment of clean reads to the reference genome yielded uniquely mapped read rates of 87.75%-90.29% (**Supplementary Table 2**), indicating that the sequencing data were suitable for downstream analyses. Correlation analysis showed that all biological replicates within each sample group were highly consistent, with correlation coefficients greater than 0.94 (**Supplementary Figure 2A**). PCA further confirmed that biological replicates clustered closely within groups, indicating high reproducibility (**Supplementary Figure 2B**). In addition, qRT-PCR analysis of 10 selected genes showed a significant positive correlation with the RNA-seq results (R = 0.90, P < 0.01; **Supplementary Figure 3**), further supporting the reliability of the transcriptomic data.

### Expression patterns and functional enrichment analysis of differentially expressed genes across developmental stages

**Figure 1:**
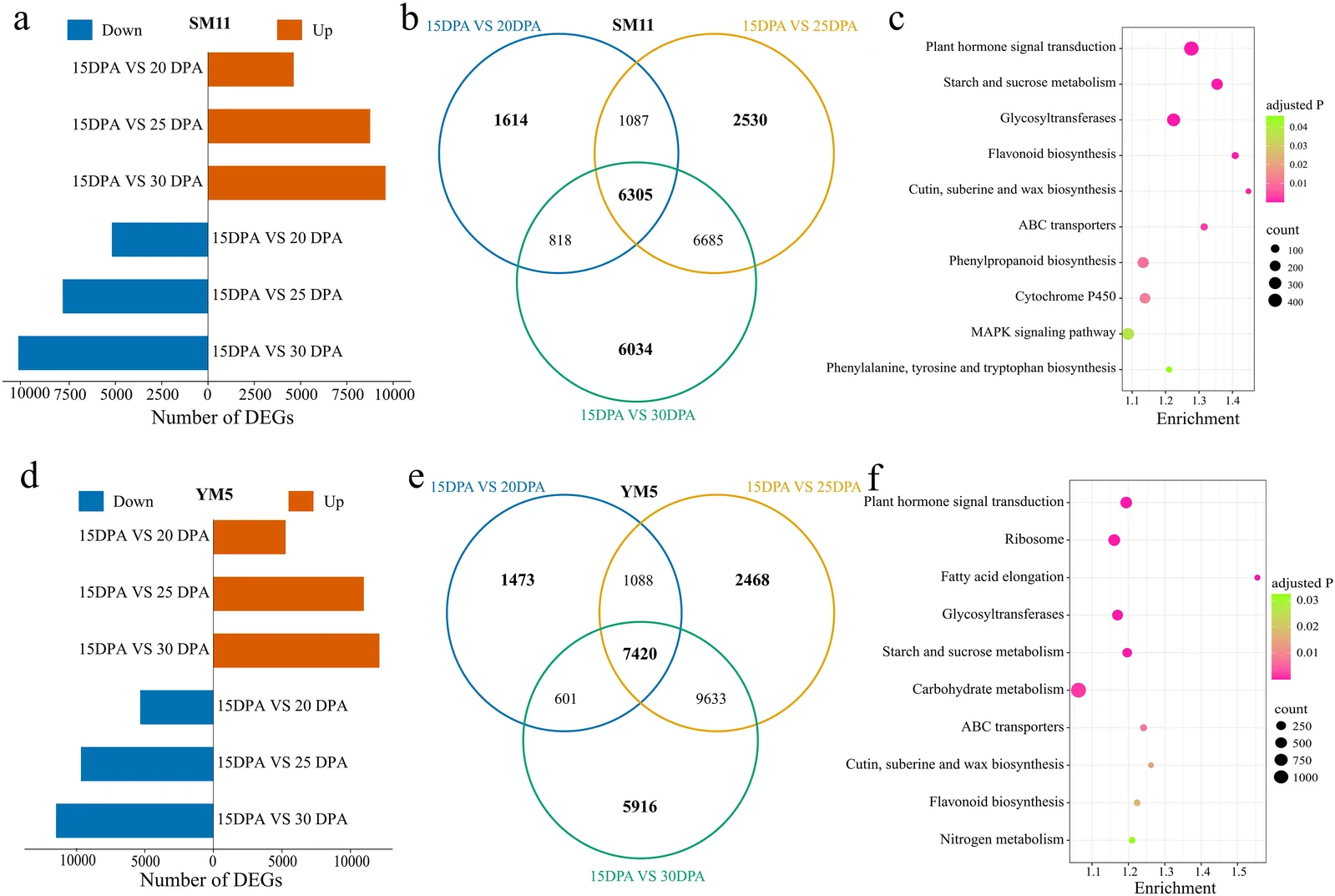
Dynamic changes and KEGG enrichment analysis of DEGs during fiber development in SM11 and YM5. **(A)** Numbers of upregulated and downregulated DEGs in SM11 at 20, 25, and 30 DPA relative to 15 DPA. **(B)** Venn diagram showing shared and stage-specific DEGs among the three comparisons in SM11: 15 DPA vs 20 DPA, 15 DPA vs 25 DPA, and 15 DPA vs 30 DPA. **(C)** KEGG enrichment analysis of all DEGs identified in SM11 across different developmental stages. **(D)** Numbers of upregulated and downregulated DEGs in YM5 at 20, 25, and 30 DPA relative to 15 DPA. **(E)** Venn diagram showing shared and stage-specific DEGs among the three comparisons in YM5. **(F)** KEGG enrichment analysis of all DEGs identified in YM5 across different developmental stages.

Comparative transcriptomic analysis across developmental stages in SM11 identified 25,073 DEGs, of which 6,305 were shared among all pairwise comparisons (**Figures 1A, B**). Compared with 15 DPA, 9,824, 16,607, and 19,842 DEGs were identified at 20, 25, and 30 DPA, respectively, including 1,614, 2,530, and 6,034 stage-specific DEGs. Among these, 4,631 genes were upregulated and 5,193 were downregulated at 20 DPA; 8,761 were upregulated and 7,846 were downregulated at 25 DPA; and 9,590 were upregulated and 10,252 were downregulated at 30 DPA. The progressive increase in DEG number during development suggests increasingly extensive transcriptional reprogramming at later stages of fiber development in SM11. KEGG enrichment analysis showed that these DEGs were mainly enriched in plant hormone signal transduction, starch and sucrose metabolism, glycosyltransferases, flavonoid biosynthesis, cutin, suberin and wax biosynthesis, ABC transporters, phenylpropanoid biosynthesis, cytochrome P450, MAPK signaling pathway, and phenylalanine, tyrosine and tryptophan biosynthesis (**Figure 1C**).

In YM5, comparative transcriptomic analysis identified a total of 28,599 DEGs, including 7,420 shared across all comparisons (**Figures 1D, E**). Compared with 15 DPA, 10,582, 20,609, and 23,570 DEGs were detected at 20, 25, and 30 DPA, respectively, with 1,473, 2,468, and 5,916 being stage-specific. Of these, 5,257 genes were upregulated and 5,325 were downregulated at 20 DPA; 10,962 were upregulated and 9,647 were downregulated at 25 DPA; and 12,099 were upregulated and 11,471 were downregulated at 30 DPA. Similar to SM11, the number of DEGs in YM5 increased progressively as development proceeded, indicating extensive transcriptional regulation during the middle and late stages of fiber development. KEGG enrichment analysis indicated that these DEGs were primarily enriched in plant hormone signal transduction, ribosome, fatty acid elongation, glycosyltransferases, starch and sucrose metabolism, carbohydrate metabolism, ABC transporters, cutin, suberin and wax biosynthesis, flavonoid biosynthesis, and nitrogen metabolism (**Figure 1F**).

Overall, DEGs in both SM11 and YM5 were significantly enriched in plant hormone signal transduction, starch and sucrose metabolism, glycosyltransferases, ABC transporters, cutin, suberin and wax biosynthesis, and flavonoid biosynthesis, suggesting that these processes may represent a common molecular basis of cotton fiber development. However, SM11 showed stronger enrichment in phenylpropanoid biosynthesis, cytochrome P450, and MAPK signaling, whereas YM5 was more prominently enriched in ribosome-related processes, fatty acid elongation, and nitrogen metabolism, indicating distinct regulatory programs underlying fiber development in the two cultivars.

**Figure 2:**
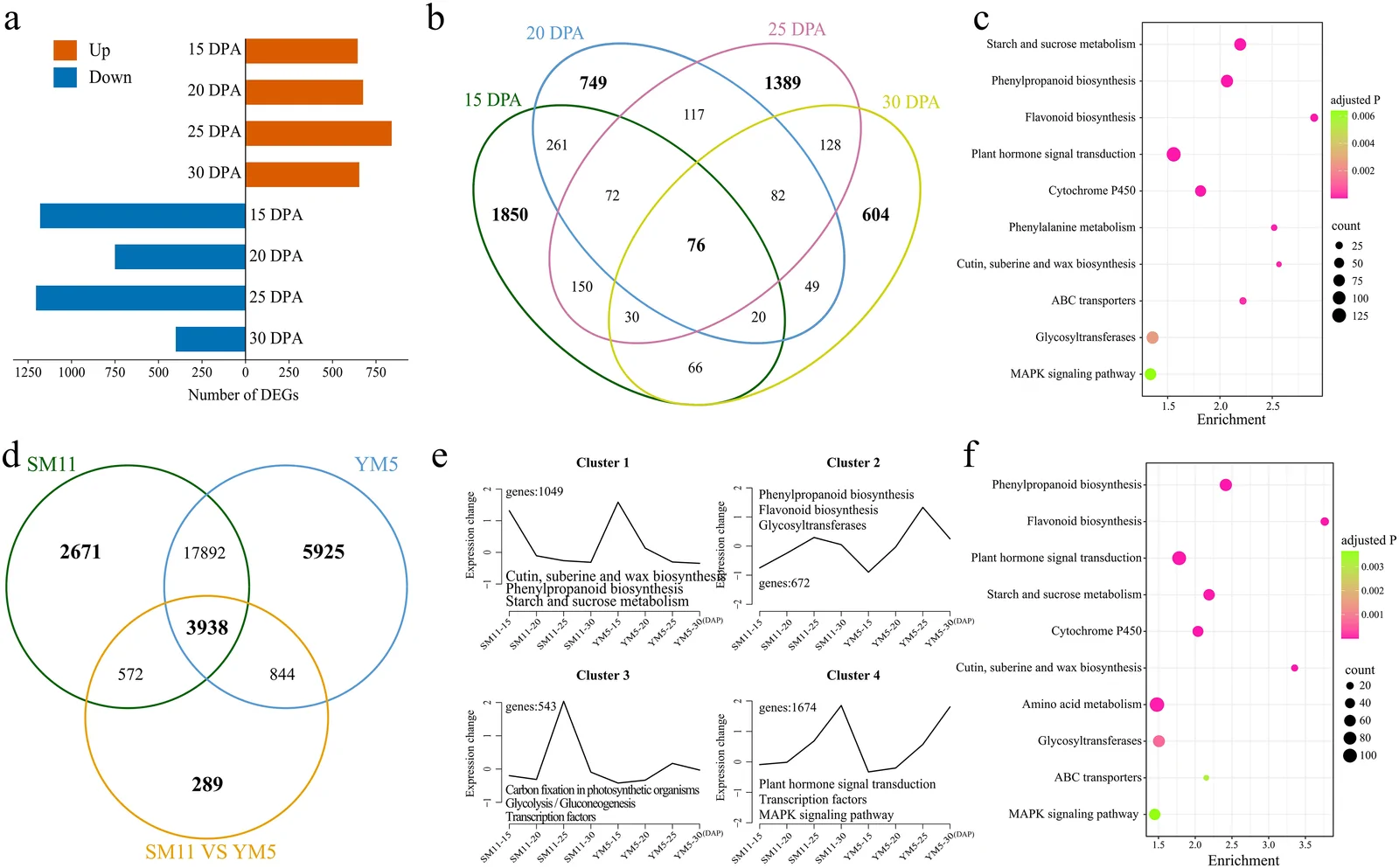
Comparative analysis and functional characterization of DEGs between SM11 and YM5 at different stages of fiber development. **(A)** Numbers of upregulated and downregulated DEGs between SM11 and YM5 at the same fiber developmental stages (15, 20, 25, and 30 DPA). **(B)** Venn diagram showing shared and stage-specific DEGs among the different developmental stages. **(C)** KEGG enrichment analysis of all DEGs identified from the pairwise comparisons between SM11 and YM5 at the same developmental stages. **(D)** Venn diagram showing shared and unique DEGs identified from comparisons across different developmental stages in SM11, across different developmental stages in YM5, and between SM11 and YM5 at matched developmental stages. **(E)** Four temporal expression patterns of the 3,938 shared DEGs identified by k-means clustering analysis. **(F)** KEGG enrichment analysis of the shared DEGs.

To further characterize transcriptional divergence between SM11 and YM5 at corresponding developmental stages, 5,644 non-redundant DEGs were identified, of which 76 were shared across all four stages (**Figures 2A, B**). Stage-matched comparisons detected 2,525, 1,426, 2,044, and 1,055 DEGs at 15, 20, 25, and 30 DPA, respectively. Among these, 644, 676, 840, and 654 genes were upregulated, whereas 1,811, 750, 1,204, and 401 were downregulated. The numbers of stage-specific DEGs were 1,850, 749, 1,389, and 604, respectively. KEGG enrichment analysis showed that these DEGs were mainly enriched in starch and sucrose metabolism, phenylpropanoid biosynthesis, flavonoid biosynthesis, plant hormone signal transduction, cytochrome P450, phenylalanine metabolism, cutin, suberin and wax biosynthesis, ABC transporters, glycosyltransferases, and MAPK signaling (**Figure 2C**), suggesting that sugar metabolism, secondary metabolism, and hormone signaling contribute to the differential fiber development observed between the two cultivars.

Among the 32,131 total DEGs identified, 2,671 were specific to SM11, 5,925 to YM5, and 289 to direct stage-matched comparisons between the two cultivars, whereas 3,938 were shared across the datasets (**Figure 2D**). Based on k-means clustering analysis, the 3,938 shared DEGs were classified into four clusters with distinct expression patterns (**Figure 2E**). Cluster 1 comprised 1,049 DEGs that were generally downregulated during fiber development in both SM11 and YM5, and were mainly enriched in pathways related to cutin, suberine and wax biosynthesis, phenylpropanoid biosynthesis, and starch and sucrose metabolism. Cluster 2 included 672 DEGs that showed a gradual upregulation trend in SM11 but decreased at the later developmental stage in YM5, with enrichment in phenylpropanoid biosynthesis, flavonoid biosynthesis, and glycosyltransferase-related pathways. Cluster 3 contained 543 DEGs that exhibited stage-specific upregulation in SM11, whereas their expression changed relatively little in YM5; these genes were mainly associated with carbon fixation in photosynthetic organisms, glycolysis/gluconeogenesis, and transcription factors. Cluster 4, the largest cluster, comprised 1,674 DEGs that were generally upregulated during fiber development in both genotypes and were enriched in plant hormone signal transduction, transcription factors, and the MAPK signaling pathway. Together, these results indicate that the shared DEGs display pronounced developmental stage- and genotype-associated expression differences during fiber development, suggesting that metabolic processes, hormone signaling, and transcriptional regulation may contribute to the differences in fiber development between SM11 and YM5. KEGG enrichment analysis of these shared DEGs revealed significant enrichment in phenylpropanoid biosynthesis, flavonoid biosynthesis, plant hormone signal transduction, starch and sucrose metabolism, cytochrome P450, cutin, suberin and wax biosynthesis, amino acid metabolism, glycosyltransferases, ABC transporters, and MAPK signaling (**Figure 2F**). Together, these results suggest that differences in cell wall-related metabolism, secondary metabolism, and hormone-associated processes underlie the divergent fiber developmental programs of the two cultivars.

### Proteomic expression characteristics and analysis of differentially accumulated proteins

**Figure 3:**
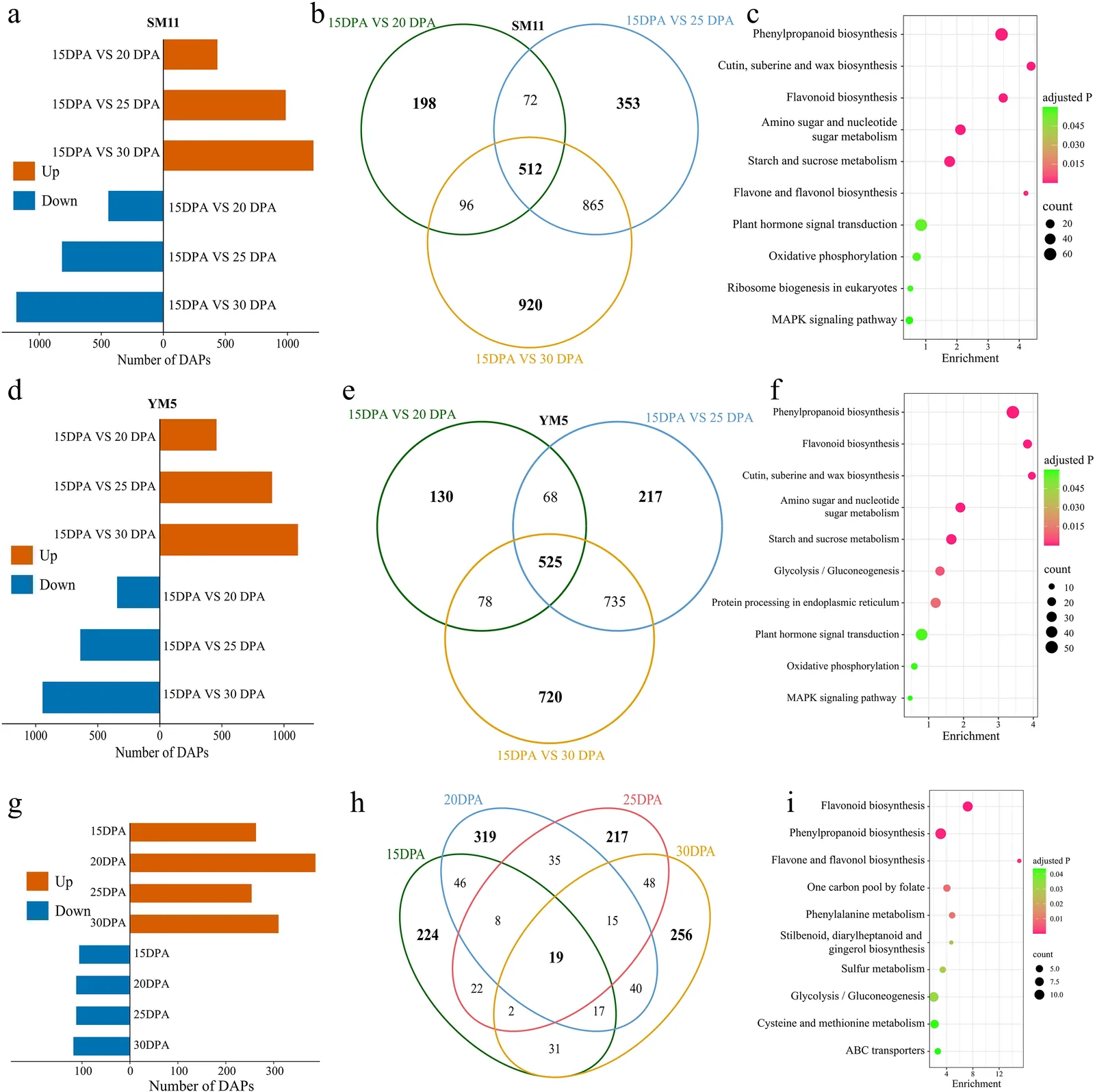
Dynamic changes and functional enrichment analysis of DAPs during fiber development in SM11 and YM5. **(A)** Numbers of upregulated and downregulated DAPs in SM11 at 20, 25, and 30 DPA relative to 15 DPA. **(B)** Venn diagram showing shared and stage-specific DAPs in SM11 among the comparisons of 15 DPA vs 20 DPA, 15 DPA vs 25 DPA, and 15 DPA vs 30 DPA. **(C)** KEGG enrichment analysis of DAPs identified in SM11 across different developmental stages. **(D)** Numbers of upregulated and downregulated DAPs in YM5 at 20, 25, and 30 DPA relative to 15 DPA. **(E)** Venn diagram showing shared and stage-specific DAPs in YM5 among the comparisons of 15 DPA vs 20 DPA, 15 DPA vs 25 DPA, and 15 DPA vs 30 DPA. **(F)** KEGG enrichment analysis of DAPs identified in YM5 across different developmental stages. **(G)** Numbers of upregulated and downregulated DAPs identified from comparisons between SM11 and YM5 at the same developmental stages (15, 20, 25, and 30 DPA). **(H)** Venn diagram showing shared and stage-specific DAPs identified from comparisons between SM11 and YM5 at the same developmental stages. **(I)** KEGG enrichment analysis of DAPs identified from comparisons between SM11 and YM5 at the same developmental stages.

To characterize dynamic changes in protein abundance during cotton fiber development, comparative proteomic analyses were performed on 24 fiber samples collected from SM11 and YM5 at 15, 20, 25, and 30 DPA. PCA showed tight clustering of biological replicates within each group, indicating high reproducibility of the proteomic dataset (**Supplementary Figure 4**). In SM11, relative to 15 DPA, 878, 1,802, and 2,393 DAPs were identified at 20, 25, and 30 DPA, respectively, with 512 shared across the three stages (**Figures 3A, B**). The number of DAPs increased progressively with development, and upregulated proteins outnumbered downregulated proteins at both 25 and 30 DPA. These DAPs were mainly enriched in phenylpropanoid biosynthesis, cutin, suberin and wax biosynthesis, flavonoid biosynthesis, amino sugar and nucleotide sugar metabolism, starch and sucrose metabolism, flavone and flavonol biosynthesis, plant hormone signal transduction, oxidative phosphorylation, ribosome biogenesis in eukaryotes, and MAPK signaling (**Figure 3C**).

In YM5, 801, 1,545, and 2,058 DAPs were identified at 20, 25, and 30 DPA, respectively, compared with 15 DPA, among which 525 were shared across the three stages (**Figures 3D, E**). As in SM11, the number of DAPs increased progressively during development, indicating more extensive proteomic reprogramming at later stages. Functional enrichment analysis showed that these DAPs were mainly enriched in phenylpropanoid biosynthesis, flavonoid biosynthesis, cutin, suberin and wax biosynthesis, amino sugar and nucleotide sugar metabolism, starch and sucrose metabolism, glycolysis/gluconeogenesis, protein processing in the endoplasmic reticulum, plant hormone signal transduction, oxidative phosphorylation, and MAPK signaling (**Figure 3F**).

Stage-matched comparisons between SM11 and YM5 identified 1,299 non-redundant DAPs, of which only 19 were shared across all four developmental stages (**Figures 3G, H**). At 15, 20, 25, and 30 DPA, 369, 484, 381, and 428 DAPs were detected, respectively, with the largest number observed at 20 DPA, suggesting that this stage represents the greatest divergence in protein accumulation between the two cultivars. KEGG enrichment analysis of these stage-matched DAPs revealed significant enrichment in flavonoid biosynthesis, phenylpropanoid biosynthesis, flavone and flavonol biosynthesis, one carbon pool by folate, phenylalanine metabolism, stilbenoid, diarylheptanoid and gingerol biosynthesis, sulfur metabolism, glycolysis/gluconeogenesis, cysteine and methionine metabolism, and ABC transporters (**Figure 3I**). Together, these results suggest that phenylpropanoid/flavonoid metabolism, carbohydrate metabolism, and epidermis- and cell wall-related processes are closely associated with the developmental divergence between the two cultivars.

### Analysis of differentially accumulated metabolites and metabolic pathway enrichment

To characterize dynamic metabolic changes during cotton fiber development, metabolomic profiling was performed on 48 fiber samples collected from SM11 and YM5 at 15, 20, 25, and 30 DPA using UPLC-MS, through which 1,995 metabolites were identified. PCA showed tight clustering of biological replicates within each group, indicating high reliability and reproducibility of the metabolomic dataset (**Supplementary Figure 5**). These metabolites were further classified into 16 major categories, among which lipids and lipid-like molecules, organic acids and derivatives, and organoheterocyclic compounds were the most abundant (**Supplementary Figure 5**).

**Figure 4:**
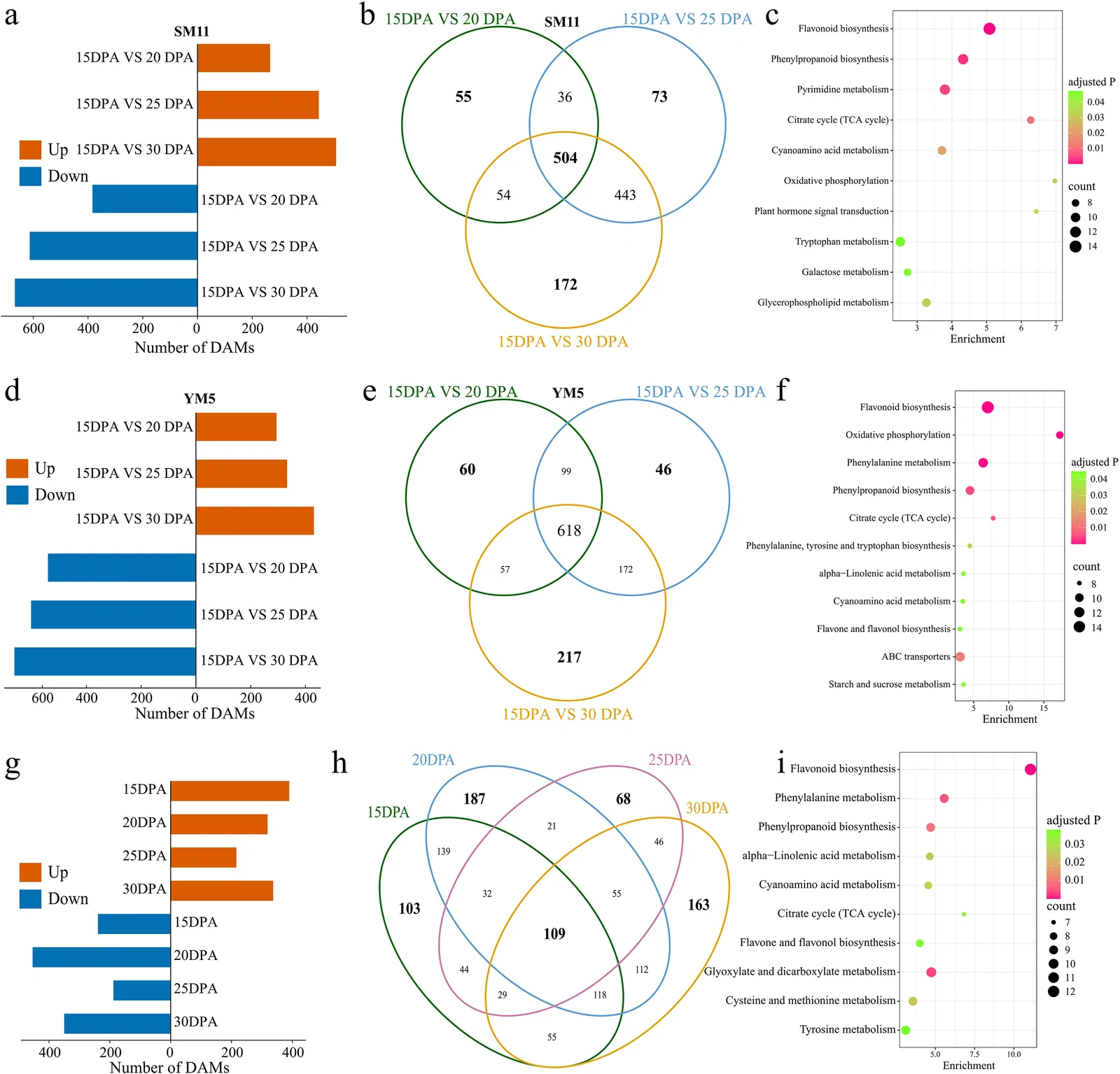
Dynamic changes and KEGG enrichment analysis of DAMs during fiber development in SM11 and YM5. **(A)** Numbers of up-accumulated and down-accumulated DAMs in SM11 at 20, 25, and 30 DPA relative to 15 DPA. **(B)** Venn diagram showing shared and stage-specific DAMs in SM11 among the comparisons of 15 DPA vs 20 DPA, 15 DPA vs 25 DPA, and 15 DPA vs 30 DPA. **(C)** KEGG enrichment analysis of DAMs identified in SM11 across different developmental stages. **(D)** Numbers of up-accumulated and down-accumulated DAMs in YM5 at 20, 25, and 30 DPA relative to 15 DPA. **(E)** Venn diagram showing shared and stage-specific DAMs in YM5 among the comparisons of 15 DPA vs 20 DPA, 15 DPA vs 25 DPA, and 15 DPA vs 30 DPA. **(F)** KEGG enrichment analysis of DAMs identified in YM5 across different developmental stages. **(G)** Numbers of up-accumulated and down-accumulated DAMs identified from comparisons between SM11 and YM5 at the same developmental stages (15, 20, 25, and 30 DPA). **(H)** Venn diagram showing shared and stage-specific DAMs identified from comparisons between SM11 and YM5 at the same developmental stages. **(I)** KEGG enrichment analysis of DAMs identified from comparisons between SM11 and YM5 at the same developmental stages.

In SM11, relative to 15 DPA, 649, 1,056, and 1,173 DAMs were identified at 20, 25, and 30 DPA, respectively, with 504 shared across the three stages (**Figures 4A, B**). The number of DAMs increased progressively during development, and down-accumulated metabolites predominated overall, indicating substantial metabolic reprogramming at the middle and late stages of fiber development. These DAMs were mainly enriched in flavonoid biosynthesis, phenylpropanoid biosynthesis, pyrimidine metabolism, the citrate cycle (TCA cycle), cyanoamino acid metabolism, oxidative phosphorylation, plant hormone signal transduction, tryptophan metabolism, galactose metabolism, and glycerophospholipid metabolism (**Figure 4C**), suggesting important roles for secondary metabolism, energy metabolism, and hormone signaling during fiber development in SM11.

In YM5, compared with 15 DPA, 834, 935, and 1,064 DAMs were identified at 20, 25, and 30 DPA, respectively, among which 618 were shared across the three stages (**Figures 4D, E**). Similar to SM11, DAM abundance increased during development, with down-accumulated metabolites again predominating, indicating extensive metabolite remodeling at later stages. Functional enrichment analysis showed that these DAMs were mainly enriched in flavonoid biosynthesis, oxidative phosphorylation, phenylalanine metabolism, phenylpropanoid biosynthesis, the citrate cycle (TCA cycle), phenylalanine, tyrosine and tryptophan biosynthesis, alpha-linolenic acid metabolism, cyanoamino acid metabolism, flavone and flavonol biosynthesis, ABC transporters, and starch and sucrose metabolism (**Figure 4F**). These results suggest that phenylpropanoid/flavonoid metabolism, carbon metabolism, and lipid metabolism jointly contribute to metabolic regulation during fiber development in YM5.

Stage-matched comparisons between SM11 and YM5 revealed large numbers of DAMs at all four developmental stages, with the greatest divergence observed at 20 DPA and relatively fewer differences at 25 DPA (**Figure 4G**). Venn analysis showed that only 109 DAMs were shared across 15, 20, 25, and 30 DPA, whereas each stage contained distinct sets of stage-specific metabolites (**Figure 4H**), indicating strong stage specificity in metabolic divergence between the two cultivars. KEGG enrichment analysis of these stage-matched DAMs revealed significant enrichment in flavonoid biosynthesis, phenylalanine metabolism, phenylpropanoid biosynthesis, alpha-linolenic acid metabolism, cyanoamino acid metabolism, the citrate cycle (TCA cycle), flavone and flavonol biosynthesis, glyoxylate and dicarboxylate metabolism, cysteine and methionine metabolism, and tyrosine metabolism (**Figure 4I**). Together, these findings indicate that phenylpropanoid/flavonoid metabolism, amino acid metabolism, and organic acid- and lipid-related pathways contribute substantially to the metabolic divergence between the two cultivars. Overall, both SM11 and YM5 exhibited pronounced metabolic remodeling during fiber development, but differed in the magnitude of their responses and the pathways enriched. In particular, metabolites associated with phenylpropanoid metabolism, flavonoid biosynthesis, sugar metabolism, and lipid metabolism may be closely linked to cultivar-specific late-stage fiber development.

### Integrated transcriptomic and metabolomic analysis

**Figure 5:**
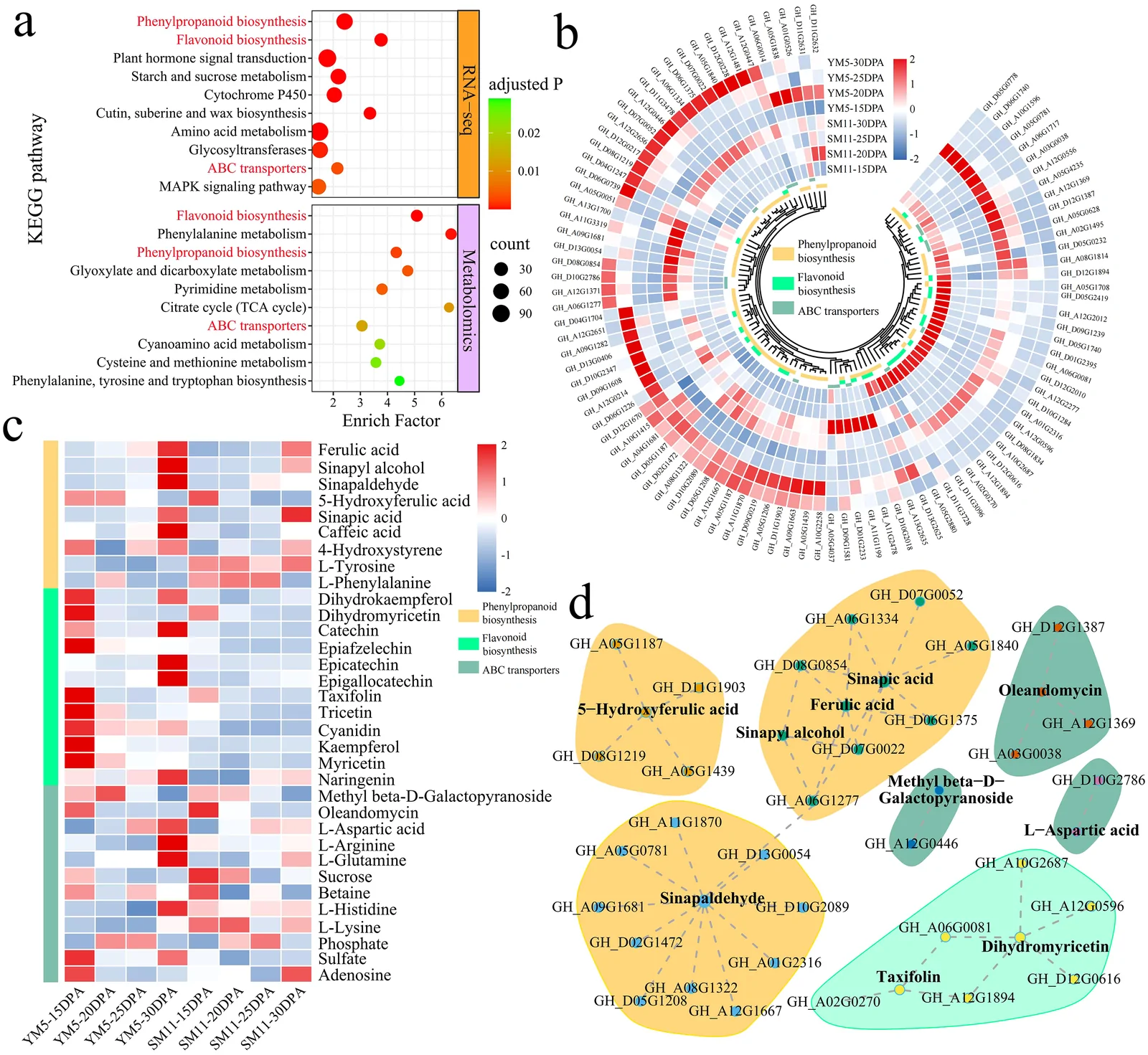
Integrated transcriptomic and metabolomic analyses reveal coordinated regulation in cotton fiber development. **(A)** KEGG enrichment analysis of the RNA-seq and metabolomic datasets. Red indicates the major pathways significantly enriched in both omics datasets. **(B)** Circular heatmap of DEGs involved in the commonly enriched pathways. Different colors indicate the pathway assignment of each gene, and the heatmap color represents the standardized expression level of each gene across different samples. **(C)** Heatmap of DAMs involved in the commonly enriched pathways. Color intensity represents the relative accumulation level of each metabolite across different samples, and different color bars indicate the pathway assignment of each metabolite. **(D)** Gene-metabolite association network constructed based on Pearson correlation analysis.

To systematically elucidate the coordinated transcriptional and metabolic changes during cotton fiber development, an integrated analysis of DEGs and DAMs was performed. KEGG enrichment analysis showed that both transcriptomic and metabolomic datasets were significantly enriched in phenylpropanoid biosynthesis, flavonoid biosynthesis, and ABC transporters, indicating that these pathways represent major processes coordinately altered at both omics levels (**Figure 5A**). Hierarchical clustering further revealed distinct accumulation patterns of candidate genes and key metabolites between SM11 and YM5 and across developmental stages, with particularly pronounced changes at 30 DPA in SM11 (**Figures 5B, C**). Pearson correlation analysis was then conducted using DEGs and DAMs involved in the commonly enriched pathways. Significant correlations were defined by q < 0.05 and |r| > 0.90, and a gene-metabolite correlation network was constructed accordingly (**Figure 5D**). Within the phenylpropanoid biosynthesis pathway, key metabolites including ferulic acid, sinapyl alcohol, sinapaldehyde, 5-hydroxyferulic acid, and sinapic acid showed significant correlations with multiple candidate genes. Similarly, flavonoid biosynthesis-related metabolites, such as taxifolin and dihydromyricetin, also exhibited strong correlations with a set of candidate genes. Several potential hub genes were identified, including *GH_A05G1187, GH_A05G1439, GH_D11G1903, GH_D08G1219, GH_A05G1840, GH_D07G0022, GH_A06G1334, GH_D06G1375, GH_A06G0081*, and *GH_A12G0446*. Together, these results indicate that phenylpropanoid metabolism, flavonoid biosynthesis, and ABC transporter-related processes form major cross-omics regulatory modules during the later stages of fiber development and may underlie the developmental divergence observed between the two cotton cultivars.

### Integrated transcriptomic and proteomic analysis

**Figure 6:**
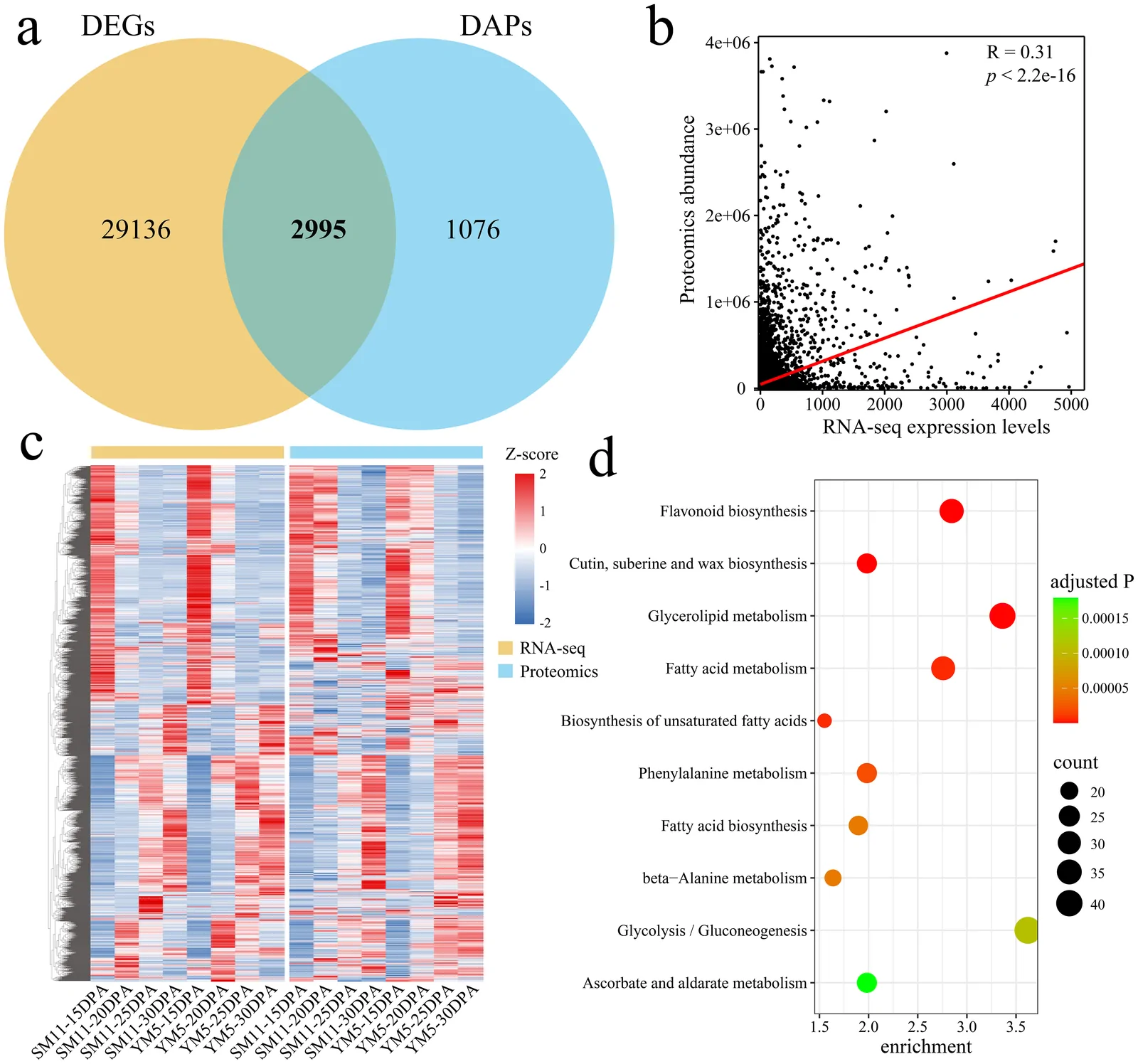
Integrated transcriptomic and proteomic analyses reveal the expression patterns and functional enrichment characteristics of shared differential molecules. **(A)** Venn diagram of DEGs and DAPs. **(B)** Correlation analysis of the shared differential molecules identified in the transcriptomic and proteomic datasets. The x-axis represents RNA-seq expression levels, and the y-axis represents protein abundance. **(C)** Heatmap showing the expression patterns of the 2,995 shared differential molecules across different cultivars and developmental stages. The left panel shows the RNA-seq expression profiles, and the right panel shows the proteomic abundance profiles; color indicates the normalized relative expression level. **(D)** KEGG enrichment analysis of the 2,995 shared differential molecules.

To further elucidate coordinated changes between the transcriptome and proteome, an integrated analysis of DEGs and DAPs was performed. Venn analysis identified 2,995 differential molecules shared between the transcriptomic and proteomic datasets, whereas 29,136 and 1,076 were unique to the transcriptome and proteome, respectively (**Figure 6A**). Correlation analysis of the shared differential molecules revealed a significant positive correlation between RNA-seq and proteomic quantification results (R = 0.31, *P* < 0.01; **Figure 6B**), indicating partial concordance between the two molecular layers. Clustering analysis of these 2,995 shared molecules further revealed dynamic expression patterns across cultivars and developmental stages (**Figure 6C**). Coordinated upregulation or downregulation was observed at specific stages, although clear discrepancies between transcript and protein abundance were also evident. These results suggest that cotton fiber development is regulated not only at the transcriptional level but also by additional post-transcriptional and post-translational mechanisms. KEGG enrichment analysis showed that the 2,995 shared differential molecules were mainly enriched in flavonoid biosynthesis, cutin, suberin and wax biosynthesis, glycerolipid metabolism, fatty acid metabolism, biosynthesis of unsaturated fatty acids, phenylalanine metabolism, fatty acid biosynthesis, beta-alanine metabolism, glycolysis/gluconeogenesis, and ascorbate and aldarate metabolism (**Figure 6D**). These pathways are primarily associated with secondary metabolism, lipid metabolism, carbon metabolism, and epidermis-related biosynthesis, indicating that coordinated transcriptome-proteome changes during cotton fiber development are concentrated in key processes related to cell wall and epidermal structure formation, membrane lipid remodeling, and energy metabolism. In particular, the enrichment of flavonoid biosynthesis, cutin, suberin and wax biosynthesis, and multiple fatty acid-related pathways suggests that coordinated regulation of secondary and lipid metabolism may contribute substantially to cultivar-specific fiber quality differences.

### Integrated transcriptomic, proteomic, and metabolomic analysis

**Figure 7:**
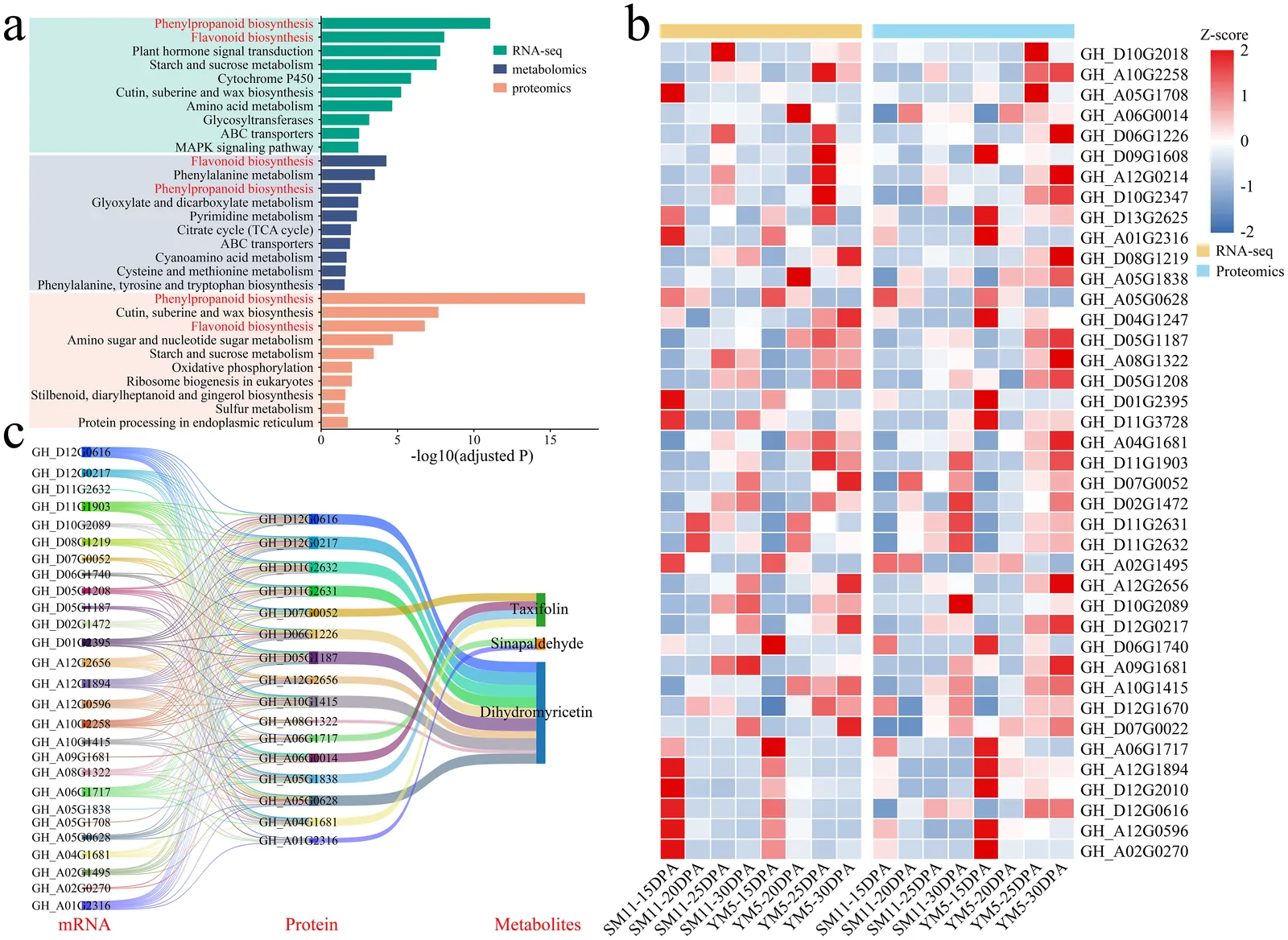
Integrated transcriptomic, proteomic, and metabolomic analyses reveal coordinated regulation of phenylpropanoid metabolism and flavonoid biosynthesis during cotton fiber development. **(A)** KEGG enrichment analysis of differential molecules identified from the transcriptomic, metabolomic, and proteomic datasets. Pathways significantly enriched in all three omics datasets are highlighted in red. **(B)** Heatmap showing the expression patterns of candidate genes involved in the commonly enriched pathways in the transcriptomic and proteomic datasets. The left panel shows the RNA-seq expression profiles, and the right panel shows the proteomic abundance profiles; color indicates the normalized relative expression level. **(C)** mRNA-protein-metabolite association network constructed based on the commonly enriched pathways. Differentially expressed genes are shown on the left, the corresponding differentially accumulated proteins in the middle, and key differentially accumulated metabolites on the right; connecting lines indicate the associations among molecules across the three omics layers.

To systematically dissect coordinated molecular changes across different regulatory layers during cotton fiber development, integrated analyses of transcriptomic, proteomic, and metabolomic datasets were performed. KEGG enrichment analysis showed that phenylpropanoid biosynthesis and flavonoid biosynthesis were significantly enriched across all three omics datasets (**Figure 7A**). Further integration of candidate molecules involved in these shared pathways revealed cultivar- and stage-dependent expression patterns at both the transcript and protein levels (**Figure 7B**). Some genes and proteins accumulated preferentially during the later stages of fiber development, whereas others exhibited opposite or asynchronous patterns between the two cultivars, indicating temporal divergence in the regulation of these key pathways. Based on these results, an integrated mRNA-protein-metabolite association network was constructed (**Figure 7C**). Several candidate genes showed coordinated or similar trends at the protein level and were closely associated with key metabolites, including taxifolin, dihydromyricetin, and sinapaldehyde. Notably, sinapaldehyde, a key intermediate of the phenylpropanoid pathway, together with taxifolin and dihydromyricetin, important products of flavonoid metabolism, exhibited accumulation patterns highly consistent with the coordinated changes of the corresponding structural genes and proteins. Together, these results identify phenylpropanoid metabolism and flavonoid biosynthesis as major cross-omics regulatory modules associated with cotton fiber development and provide candidate genes, proteins, and metabolites for further functional investigation.

## Discussion

Integrated transcriptomic, proteomic, and metabolomic analyses of fibers from SM11 and YM5 across 15–30 DPA indicate that developmental divergence between the two cultivars is unlikely to result from disruption of a single pathway, but rather from coordinated remodeling across multiple regulatory layers during a critical developmental window. Across all three omics datasets, late fiber development was characterized by strong stage-dependent reprogramming, particularly in plant hormone signal transduction, starch and sucrose metabolism, phenylpropanoid metabolism, flavonoid biosynthesis, lipid- and epidermis-related metabolism, and ABC transporter-associated processes. Although these pathways were shared between the two cultivars, their magnitude and temporal dynamics differed markedly, suggesting that cultivar-specific fiber developmental outcomes are shaped less by pathway identity than by differences in the timing, amplitude, and coordination of core biological programs. Notably, 20 DPA emerged as a particularly informative stage, at which stage-matched comparisons revealed pronounced divergence in both protein accumulation and metabolic profiles between SM11 and YM5 (Zhang et al., 2022; Grover et al., 2025). This finding is consistent with previous studies showing that cotton fibers undergo a developmental transition from rapid elongation to secondary wall deposition at approximately 15–20 DPA, a process accompanied by reduced cell expansion, activation of cell wall biosynthesis, and extensive metabolic reorganization (Qiao et al., 2025; Bai and Scheffler, 2024; Jareczek et al., 2023). Whether this stage represents a decisive developmental boundary underlying fiber quality divergence, however, remains to be determined by targeted temporal and functional analyses. Because SM11 and YM5 are not near-isogenic lines, the observed multi-omics differences may reflect both fiber-quality-associated regulatory divergence and broader cultivar-specific genetic background effects. Therefore, the candidate pathways and molecules identified here should be further validated in additional populations or near-isogenic materials.

Our results further underscore the importance of carbohydrate metabolism, lipid metabolism, and epidermis-associated biosynthetic processes during cotton fiber development. Differential enrichment of starch and sucrose metabolism, glycolysis, fatty acid metabolism, glycerolipid metabolism, and cutin/suberin/wax biosynthesis between the two cultivars indicates that fiber development is closely coupled to carbon allocation, membrane remodeling, and deposition of surface-associated structural components. This interpretation is consistent with previous studies showing that sugar metabolism regulates reactive oxygen species (ROS) homeostasis and the timing of secondary wall deposition, whereas very-long-chain fatty acid (VLCFA) biosynthesis, membrane lipid homeostasis, and cuticle-related processes are linked to fiber elongation, cell surface properties, and developmental phase transitions (Ding et al., 2021; Yang et al., 2023; Duan et al., 2023). Together, these observations support the view that sugar- and lipid-related pathways provide both the metabolic resources and structural basis required for the transition from rapid elongation to secondary wall thickening and maturation (Wen et al., 2024; Chen et al., 2023).

Cross-omics enrichment was also consistently observed in pathways related to plant hormone signal transduction and ABC transporters, suggesting that divergence between the two cultivars is shaped not only by metabolic reprogramming, but also by coordinated changes in signaling and transmembrane transport (Bai and Scheffler, 2024; Yang et al., 2023). Cotton fiber development is known to be jointly regulated by multiple phytohormones, including auxin, gibberellin, brassinosteroids, and ethylene (Bai and Scheffler, 2024; Yang et al., 2023). These hormones do not act independently; rather, they collectively influence developmental trajectories by modulating sugar utilization, cell wall biosynthesis, lipid remodeling, and ROS homeostasis (Wu et al., 2022; Biała-Leonhard et al., 2025). By contrast, the roles of ABC transporters in cotton fiber development remain relatively underexplored (Wu et al., 2022; Biała-Leonhard et al., 2025). Their repeated identification across all three omics layers in the present study raises the possibility that they mediate the transmembrane transport of flavonoids, phenolic compounds, lipid precursors, or epidermis-associated components, thereby contributing to metabolite partitioning and cell surface assembly during later stages of fiber development (Manzoor et al., 2023). Although direct functional evidence is still limited, these results support the view that transport processes represent an integral component of fiber quality divergence.

Another noteworthy finding was the stronger enrichment of phenylpropanoid metabolism, cytochrome P450, and MAPK signaling in SM11, whereas YM5 was more strongly associated with ribosome-related processes, fatty acid elongation, and nitrogen metabolism. These pathway preferences are broadly consistent with previous studies showing that fiber development depends on coordinated shifts in signaling, secondary metabolism, and lipid-associated biosynthetic programs across developmental stages (Zhang et al., 2022; Liu et al., 2025; Shi et al., 2024). SM11 appears to rely more heavily on signaling responsiveness, secondary metabolism, and cell wall-associated remodeling, whereas YM5 appears to prioritize protein biosynthetic capacity, lipid elongation, and basal nitrogen metabolism. Such divergence may reflect alternative molecular routes by which different genetic backgrounds support fiber development. More broadly, these results reinforce the view that cultivar-specific differences in fiber quality are unlikely to arise from the action of a single pathway, but instead reflect the integrated effects of multiple pathways that differ in temporal coordination, metabolic priority, and regulatory mode (You et al., 2023; Sun et al., 2025).

Among the pathways identified in this study, phenylpropanoid metabolism and flavonoid biosynthesis showed the strongest cross-omics consistency. These pathways were repeatedly enriched in both developmental-stage comparisons and stage-matched comparisons between cultivars, and were detected across the transcriptomic, proteomic, and metabolomic datasets, suggesting their close association with late-stage metabolic remodeling and cultivar-specific divergence during fiber development (Li et al., 2020; Wang et al., 2022; Shi et al., 2024; Hinchliffe et al., 2024). Consistently, association analysis linked several metabolites, including sinapaldehyde, taxifolin, and dihydromyricetin, with multiple candidate genes and proteins. The phenylpropanoid pathway provides precursors for lignin, phenolic acids, and downstream flavonoid metabolism (Xiao et al., 2014; Zheng et al., 2024; You et al., 2023), whereas flavonoid metabolism has been implicated in pigment formation, fiber elongation, redox homeostasis, and secondary wall development (Zheng et al., 2023a, b; Chen et al., 2025b). Recent evidence further indicates that the flavonoid biosynthetic enzymes GhCHS and GhDFR differentially regulate fiber elongation and secondary wall formation, respectively (Chen et al., 2026). Therefore, the recurrent enrichment of these pathways in our multi-omics analyses supports their potential involvement in the metabolic reprogramming accompanying the transition from fiber elongation to maturation (Tan et al., 2013; Li et al., 2024; Zhu et al., 2025). The coordinated variation of sinapaldehyde with associated genes and proteins may reflect changes in phenylpropanoid flux related to monolignol metabolism and secondary cell wall formation, whereas taxifolin and dihydromyricetin may be linked to flavonoid flux, redox homeostasis, and phenolic metabolic remodeling during late fiber development. These observations suggest a possible connection between precursor supply for cell wall biosynthesis and the redistribution of metabolic flux through secondary metabolic branches. However, because the current evidence is based mainly on enrichment and correlation analyses, these associations should be interpreted as hypothesis-generating rather than demonstrating direct causal regulation. Future studies should validate these candidate metabolites and their associated genes/proteins through targeted metabolite quantification, qRT-PCR or PRM assays, virus-induced gene silencing or CRISPR/Cas-mediated editing of hub genes, and exogenous application of sinapaldehyde, taxifolin, or dihydromyricetin in cotton ovule/fiber culture systems, followed by assessment of fiber length, cellulose deposition, cell wall thickness, and mature fiber quality traits.

Although the transcriptomic and proteomic datasets were significantly positively correlated, the correlation was modest (R = 0.31), indicating that transcript abundance explains only part of the variation in protein accumulation during cotton fiber development (Fan and Shen, 2025). This partial concordance is consistent with the influence of multiple regulatory layers, including mRNA stability, translational efficiency, ribosome loading, protein processing, degradation, and post-translational modifications. The enrichment of pathways related to protein processing, oxidative phosphorylation, lipid metabolism, and secondary metabolism further suggests that late fiber development involves extensive proteome remodeling beyond transcriptional regulation. Processes such as ubiquitin-proteasome or autophagy-mediated protein turnover, together with phosphorylation, acetylation, oxidation, and glycosylation, may modulate protein activity and stability independently of changes in transcript abundance. These observations highlight the importance of post-transcriptional, translational, and protein stability-related regulation in shaping cotton fiber development, and underscore why single-omics analyses may be insufficient to fully resolve its molecular basis (Grover et al., 2025). The value of multi-omics integration therefore lies not simply in increasing data volume, but in identifying cross-layer regulatory modules with stronger biological relevance. Several limitations of the present study should also be acknowledged. First, the major conclusions are based primarily on pathway enrichment, differential expression patterns, and correlation networks, which do not establish direct causal roles for the identified candidate molecules in cultivar-specific fiber divergence. Second, although the selected cultivars and sampling time points cover key stages of late fiber development, they still provide limited temporal resolution for defining the precise sequence of molecular events. Finally, the relationships among candidate metabolites, proteins, and genes remain largely statistical and require experimental validation. Future studies should therefore prioritize targeted validation of taxifolin, dihydromyricetin, sinapaldehyde, and their associated candidate genes, and assess whether these associations with fiber quality divergence remain robust across a broader range of cotton materials and genetic backgrounds.

## Conclusions

In summary, integrated transcriptomic, proteomic, and metabolomic analyses of developing fibers from SM11 and YM5 revealed extensive molecular reprogramming during the critical 15–30 DPA window, particularly in plant hormone signal transduction, starch and sucrose metabolism, cutin/suberin/wax biosynthesis, ABC transporter-associated processes, and phenylpropanoid and flavonoid metabolism. Among these pathways, phenylpropanoid biosynthesis and flavonoid biosynthesis showed the strongest cross-omics consistency, supporting their close association with cultivar-specific fiber developmental divergence. Integrative analyses further identified taxifolin, dihydromyricetin, and sinapaldehyde as key candidate metabolites associated with corresponding genes and proteins, providing potential targets for future functional validation and fiber quality improvement.

## Data Availability

The datasets presented in this study can be found in online repositories. The names of the repository/repositories and accession number(s) can be found in the article/**Supplementary Material**.

## Supplementary material

Supplementary Table 1 Sequences of all primers used in this study.

Supplementary Table 2 Summary of RNA-seq data quality and alignment statistics.

Supplementary Figure 1 Fiber quality traits of SM11 and YM5. Values are presented as mean ± SD (**P* < 0.05, ***P* < 0.01).

Supplementary Figure 2 Sample correlation and principal component analysis of RNA-seq data from SM11 and YM5 at different stages of fiber development. **(A)** Heatmap of correlations among all samples. Color indicates the strength of the correlation between samples, with green representing lower correlation and red representing higher correlation. **(B)** PCA of all samples. Each point represents one sample, and different colors indicate different developmental stages.

Supplementary Figure 3 Correlation analysis between RNA-seq and qRT-PCR results. Black dots represent individual genes, and the red line indicates the linear regression fit.

Supplementary Figure 4 PCA of proteomic samples from SM11 and YM5 at different stages of fiber development. Each point represents one sample, and different colors indicate different developmental stages.

Supplementary Figure 5 Classification and PCA of all metabolites identified in the metabolomic analysis. **(A)** Chemical classification and proportions of all detected metabolites. **(B)** PCA of metabolomic samples from SM11 and YM5 at different stages of fiber development. Each point represents one sample, and different colors indicate different genotypes and developmental stages.
